# Leaf endophyte load influences fungal garden development in leaf-cutting ants

**DOI:** 10.1186/1472-6785-12-23

**Published:** 2012-11-09

**Authors:** Sunshine A Van Bael, Catalina Estrada, Stephen A Rehner, Janette Fabiola Santos, William T Wcislo

**Affiliations:** 1 Department of Ecology and Evolutionary Biology, Tulane University, 6823 St. Charles Avenue, New Orleans, LA, 70118, USA; 2 Smithsonian Tropical Research Institute, Apartado, 0843-03092, Republic of Panama; 3 Systematic Mycology and Microbiology Laboratory, USDA-ARS, Beltsville, MD, 20705, USA

**Keywords:** *Atta colombica*, Attini, Leaf-cutting ants, Lepiotaceae, Mutualism, Symbioses

## Abstract

**Background:**

Previous work has shown that leaf-cutting ants prefer to cut leaf material with relatively low fungal endophyte content. This preference suggests that fungal endophytes exact a cost on the ants or on the development of their colonies. We hypothesized that endophytes may play a role in their host plants’ defense against leaf-cutting ants. To measure the long-term cost to the ant colony of fungal endophytes in their forage material, we conducted a 20-week laboratory experiment to measure fungal garden development for colonies that foraged on leaves with low or high endophyte content.

**Results:**

Colony mass and the fungal garden dry mass did not differ significantly between the low and high endophyte feeding treatments. There was, however, a marginally significant trend toward greater mass of fungal garden per ant worker in the low relative to the high endophyte treatment. This trend was driven by differences in the fungal garden mass per worker from the earliest samples, when leaf-cutting ants had been foraging on low or high endophyte leaf material for only 2 weeks. At two weeks of foraging, the mean fungal garden mass per worker was 77% greater for colonies foraging on leaves with low relative to high endophyte loads.

**Conclusions:**

Our data suggest that the cost of endophyte presence in ant forage material may be greatest to fungal colony development in its earliest stages, when there are few workers available to forage and to clean leaf material. This coincides with a period of high mortality for incipient colonies in the field. We discuss how the endophyte-leaf-cutter ant interaction may parallel constitutive defenses in plants, whereby endophytes reduce the rate of colony development when its risk of mortality is greatest.

## Background

Foliar endophytic fungi (hereafter “endophytes”) live within leaves and other above-ground plant tissues without causing any apparent signs of disease [1]. Previous work in temperate areas has demonstrated that some endophytes defend their host plants by making their leaves less palatable to insect herbivores [2-5]. Leaf-cutting ants (genera *Atta* and *Acromyrmex*, Myrmicinae) maintain an obligate symbiosis with their fungal cultivar (Basidiomycota, *Leucocoprinus gongylophorus*) [6,7]. The ants defoliate a wide diversity of plants and often have an enormous effect on local flora and distribution of nutrients [8-10]. The worker ants cut leaves, carry them to the nest, clean them, and use them as compost to cultivate their symbiont fungus. The ants’ fungal symbiont then partially degrades the leaf material, converting leaf biomass to fungal food for the ants.

While the ant-cultivar symbiosis is relatively well studied, the potential interactions among the ants’ cultivar and fungal endophytes are only recently receiving attention. In the temperate zone, one previous experiment with grasses found evidence of endophyte toxicity toward leaf-cutting ant queens for some grass-endophyte combinations, suggesting a defensive mutualism between the grass and endophyte [11]*.* A few descriptive studies have suggested that some fungal endophytes can enter and persist in the ant’s gardens. Endophytes were isolated from the gardens of naturally occurring *Acromyrmex* sp. nests [12], and from laboratory colonies of *Atta cephalotes*; in the latter endophyte composition changed when ants were offered a new food source [13]. Despite these observations, little is known about how ants and their fungal cultivar interact with endophytic fungi in their forage material [14-17].

Previous research with laboratory colonies of *Atta colombica* (Guérin-Méneville) (Myrmicinae; Attini) in Panama has shown that (1) ants spend 45% more time cutting and removing leaf pieces with high versus low endophyte loads, (2) ants reduce the amount of endophytic fungi in leaves before planting them in their gardens and (3) the ants’ fungal cultivar inhibits the growth of most endophytes tested using *in vitro* bioassays [14]. Moreover, when given a choice, leaf-cutter ants cut nearly 50% more leaf area from seedlings with low endophyte relative to high endophyte loads [17]. These short-term experiments, however, did not test whether the extra time spent cutting and preparing leaves translated into a cost for the colony, such as reduced fungal growth and overall colony development. The overall growth rate of the colony is likely to depend on many factors, including the maturity of the colony, environmental conditions, and the amount of time necessary for worker ants to cut, process and plant material in the fungal gardens. Moreover, the ants’ fungal cultivar is likely to interact with plant material from different host plants in idiosyncratic ways, with some host plants providing a superior substrate for cultivar growth.

Here we report a 20-week long experiment that monitored the development of *A. colombica* colonies foraging on leaves with high or low endophyte loads. The experiment was conducted to provide evidence for or against the hypothesis that *foliar endophytes reduce defoliation to plants that host them by negatively affecting leaf-cutting ant colonies and their fungal symbiont.* Previous observations with *A. colombica* suggested that endophytes were not directly toxic to the ants or their fungi (Van Bael *unpublished*). We therefore predicted that endophytes would exact a cost on ant colonies by slowing their overall development rate. This prediction assumes that early ant colony development is limited by how much leaf material the workers can cut.

An alternative hypothesis is that leaf-cutting ants and their cultivar benefit from the activity of certain endophytic fungi in their fungal gardens. In this case, leaf-cutting ants could choose to remove some strains of endophytes, but actively plant and encourage the growth of others. There may be an unrecognized benefit to maintaining diverse endophytic fungi in leaf-cutter ant gardens, as suggested for microbiome diversity in general (*e.g*., [18]). If such benefits outweigh the cost of extra time needed to manipulate endophytes, we would expect to see greater survivorship and growth rates in colonies that foraged on leaves with high endophyte loads.

## Methods

### Study site and study species

The Gamboa field station (9° 07^′^N, 79° 42^′^W) of the Smithsonian Tropical Research Institute (STRI) in the Republic of Panama borders lowland, wet tropical forest and hosts a high abundance and diversity of plants, endophytic fungi and leaf-cutting ants. The most common leaf-cutting ant species in the forest margins and open areas of Gamboa is *Atta colombica*. We grew cucumber plants (*Cucumis sativus*) from seeds and cassava plants (*Manihot esculenta*) from cuttings as both plants grew rapidly in greenhouse conditions and provided a constant source of forage material for laboratory colonies.

### Endophyte treatments

To provide E_low_ and E_high_ forage material to the feeding groups, we manipulated endophyte density and diversity of leaf material using two different techniques. Initially, plants were maintained free of endophytes by planting seeds or cuttings in sterile growth chambers (2 weeks). Then all *C. sativus* plants were moved to plastic enclosures inside of greenhouses (2–4 weeks), while *M. esculenta* plants remained in the growth chambers. We then introduced fungi in two ways. For *M. esculenta*, laboratory inoculations involved growing pure cultures of *Colletotrichum tropicale* ( [19], strain Q633) conidia in broth, concentrating conidia in sterile water, and applying them as a spray onto cassava leaves. Control plants had sterile water sprayed on their leaves (as in [14]). For *C. sativus*, forest inoculations involved moving a subset of potted cucumber plants from the greenhouse to the forest during the night time only. The plants obtained the natural complement of endophyte spore fall by night, but the daytime conditions in the growth chamber remained the same for inoculated and control plants (as in [17]) (for details on plant inoculations see Additional file 1).

Throughout our study, we isolated endophytes from 124 experimental leaves of *C. sativus* and 12 experimental leaves of *M. esculenta* to assess the abundance and diversity of endophytes and thereby check the validity of our treatments. Using standard endophyte isolation methods [20], we cut 2 mm^2^ leaf segments, surface sterilized them, and plated them on to 2% malt extract agar (MEA), a standard mycological medium. The plates were sealed and incubated at room temperature until we observed mycelia extending into the medium. From the *C. sativus* leaf pieces, a subset of the fungi (207 individual strains) were isolated into pure culture. After two weeks, plates were sorted by morphospecies and one or two individuals of the most common 5 morphotypes were identified using molecular genetic methods (n = 8 fungal strains, detailed below).

We harvested some of our treated cucumber leaves throughout the experiment for measurements of leaf mass per unit leaf area (LMA) and of nutritional content. These leaves were inoculated using forest inoculations, and were from the same plants that we used to provide forage material to the ants. For LMA leaves, we scanned the leaves to measure leaf area and dried them in a drying oven at 70°C for at least 5 days. Then we measured the mass of each dried leaf to estimate the LMA on a per leaf basis (n = 60 leaves). An additional subset of cucumber forage leaves were dried and sent for analysis of nutritional content (P, K, Ca, Mg, Zn, Mn, Cu, Fe) at the University of Florida Analytical Research Laboratory (Gainesville, FL).

### Feeding experiment

We collected ~ 450 incipient *A. colombica* queens during their nuptial flight in May 2010. *A. colombica* queens carry a small piece of their natal fungal garden for establishment after mating. We kept the queens in small plastic containers with a small amount of soil collected from areas where they were found digging. Queens naturally established their fungal gardens, produced eggs and survived this period without external nutrition provided by our experiment. After five weeks, approximately 40% of the queens survived and we photographed their growing fungal garden to assess fungal area. To minimize disturbance, we did not remove queens during these photographs. When the first workers emerged (~6 weeks after the queens’ flight) we selected 120 colonies to include in our fungal garden development experiment. Colonies were haphazardly assigned to a low endophyte diet (hereafter, E_low_, n=60) or a high endophyte diet (E_high_, n=60) group. These colonies were placed in a 15 × 30 cm open plastic container with fluon (Teflon® PTFE 30) surrounding the edges to prevent the foraging ants’ escape. This plastic container also served as the foraging arena.

Over the course of the feeding experiment, colonies were provided with (1) cucumber leaves with high or low densities of endophytes, using leaf material from forest inoculations (1 seedling /day, 5 days/week); (2) cassava leaves with high or low density endophytes, using laboratory inoculations (~1-2 leaves/month); and (3) an open petri plate with 1 g of mixed corn meal and oats (for supplementary feeding over the weekend, 2 days/week). We did not remove uncut plants or leaves from prior feedings until they were replaced with fresh plants in subsequent feedings. Our intent was to constantly provide forage material (with some diversity) to the colonies, although the natural diversity of *Atta colombica* forage material is much higher [9].

We haphazardly chose colonies in each treatment group to destructively sample after 2, 4, 10, 16 and 20 weeks of foraging. The sample sizes for E- and E+ colonies from each period were; 15 and 15 colonies in week 2, 15 and 13 colonies in week 4, 9 and 8 colonies in week 10, 9 and 9 colonies in week 16, and 7 and 9 colonies in week 20. The effort needed to produce the forage material necessitated having fewer fungal gardens as the colonies grew. At each sampling time, we placed the colonies in the freezer. We then measured the fresh weight of the entire colony (including brood, garden and all ants except for the queen), the queens’ fresh mass, and we photographed each colony. We then separated the workers from the fungal garden and counted the pupae and the worker ants, grouping them into 2 size categories; small < 3 mm, and large > 3 mm. We lyophilized the fungal garden and measured the dry mass of the fungal garden only.

### Laboratory vs. field comparisons

We wanted to assess the impact of our laboratory conditions and feeding regime on general microbial communities within the ants’ fungal garden. We sampled from 18 laboratory fungal gardens for culturable bacteria and fungi during week 16 of the feeding experiment. During the three previous weeks, we sampled from 19 *A. colombica* fungal gardens in the field around Gamboa in order to compare the microbial communities in field and laboratory conditions. Each sampling consisted of plating 18 1.5 mm^2^ pieces of the fungal garden per colony on 3 MEA plates (6 per plate). We scored the plates after 7 days to assess whether the garden isolate grew the ants’ symbiont fungi only, non-symbiont fungi, or bacteria. In some cases we saw that the fungal garden isolate produced both symbiont and non-symbiont fungi, in which case we scored them for both. We calculated percentages of fungal garden isolates with each type of growth, with overall percentages reaching over 100% in some cases where several types of fungi or bacteria grew out of the fungal garden section. Within the non-symbiont fungi we did a visual estimate of how many morphospecies per isolate were present on the plate, based on colony characteristics. Some garden pieces grew more than one non-symbiont or bacteria morphotype, in which case we counted each as an individual.

We also documented incipient ant queen and colony mortality in field versus laboratory conditions. In 2011, we measured the survival of *A. colombica* colonies in the field during 3 months after queens flew. This was measured in 4 plots that were ~ 25 m^2^ each, at least 0.5 km apart, and were all in open areas where queens dug holes in high abundance. Our first measurement was taken on the morning after the mating flight, when the queens’ entrance holes were clearly visible. The second measurement was 8 weeks later, when the young incipient colonies had just emerged. The third measurement was 4 weeks later, to emulate the 4-week sample in our laboratory feeding experiment. Across the plots we measured the fate of 375 queens.

### Molecular analyses

We selected 8 representative strains from our 5 most common morphospecies isolated from cucumber leaves as endophytes, and 13 strains isolated from the fungal gardens as non-symbiont fungi, to identify the most common morphotypes present in the leaves and gardens. To determine the taxonomic affinities of these strains, primers ITS5 and ITS4 [21] were used to amplify the approximately 540bp ITS, followed by sequencing with the same primers.

### Statistical analyses

We used general linear models to estimate the effects of endophyte treatment (nested within sampling time) on fresh colony mass, number of workers, queen mass, dry garden mass, fresh colony mass per worker (× 1000), and dry garden mass per worker (× 1000). We followed this with Mann–Whitney U tests for the individual sample times. For various reasons, such as colony mortality, queen mortality, and problems with fungal garden masses, our final sample size for most general linear models was approximately 105–108 colonies. We used standard t tests to compare LMA, nutrient content, endophyte abundance, and garden isolates between the treatments or sampling areas. These tests were all performed using Systat [22]. We further used the species diversity estimator Chao 2 [23] to compare the endophyte morphospecies diversity in our forage material.

## Results

### Endophyte treatments

Our endophyte treatments resulted in leaf tissue that had significantly different endophyte loads. Over the course of the experiment, the mean (± 1 se) % of leaf pieces from *C. sativus* with endophytes for E_low_ and E_high_ leaves were 39 ± 4% and 93 ± 2% respectively, which was significantly different (two tailed t-test, t = 12.7, 82 d.f., P < 0.001). In contrast, the estimated number of morphospecies was similar for the two treatments. We isolated 38 morphospecies (95% CI 27–49) from E_low_ leaves, and 39 morphospecies (95% CI 28–50) for E_high_ leaves. Using a species diversity estimator Chao 2, the two treatments also showed no difference between E_low_ (estimated at 134 morphospecies (95% CI 71–315)) and E_high_ (estimated at 124 morphospecies (95% CI 68–287)) leaves. Our isolations from *M. esculenta* showed that *C. tropicale* grew from 68 ± 2% and 0.1 ± .01% of the leaf pieces for E_high_ and E_low_ leaves respectively. We did not observe endophytes other than *C. tropicale* in the *M. esculenta* plants, as they were protected from ambient spore fall in growth chambers.

We sampled leaves throughout the experiment to test whether the treatments differed in several leaf parameters. We found that the two treatments did not differ with respect to mean (± 1 se) mass per unit leaf area mg cm^-2^ (E_low_ 1.14 ± 0.04, E_high_ 1.11 ± 0.04, t = −0.54, d.f. = 58, P = 0.56). Leaf tissue from the two endophyte treatments did not differ with respect to nutrients (P, K, Ca, Mg, Zn, Mn, Cu, Fe; n=6, all *P* values > 0.10, data not shown).

### Feeding experiment

At the outset of the feeding experiment, we measured the area of each fungal garden before any workers were born and foraging. After the experiment, we confirmed that initial area of the fungal garden was not different between E_low_ and E_high_ treatments (t = −0.39, d.f. = 107, P = 0.70).

Colony mortality was low throughout the experiment and did not differ among the treatments. Specifically, only 9% (11/120) of the colonies did not survive the experiment, with 4% and 5% not surviving in E_low_ and E_high_ treatment groups, respectively. Most mortality (10/11 colonies) occurred between 2 and 10 weeks of ants’ foraging.

**Figure 1:**
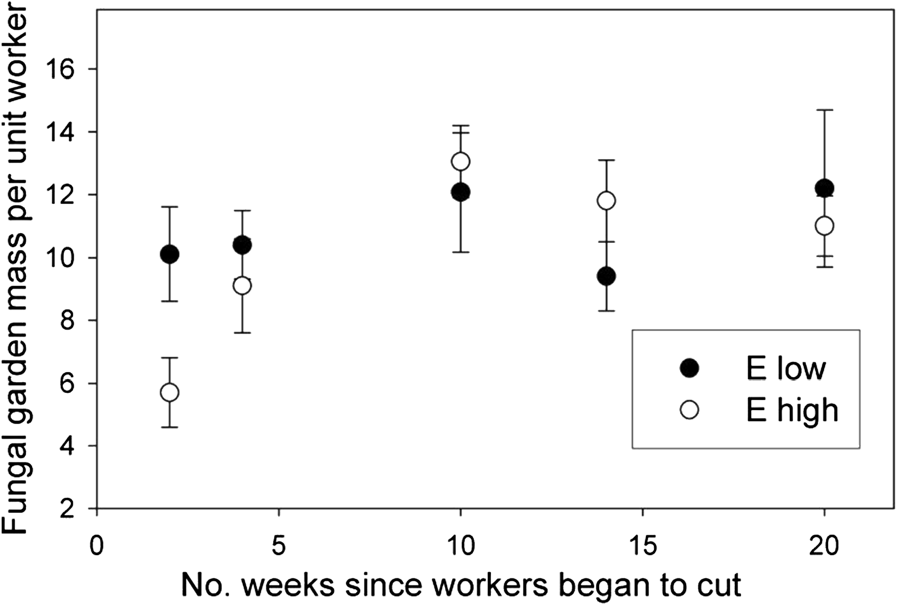
**The mean (± 1 standard error) fresh colony mass per unit worker for colonies (in grams x 1000) that were fed leaves with high (E**_**high**_**) and low (E**_**low**_**) endophyte densities.**

The results of our general linear models showed that the effect of E_low_ and E_high_ treatment on fresh fungal garden mass, dry garden mass, the total number of workers and the queen mass was not significant (Table 1). We observed a marginally significant trend toward greater values of fungal garden mass per worker in E_low_ relative to E_high_ colonies, for both fresh and dry mass measurements (Table 1). When we plotted the fungal garden mass per worker over the different harvest time periods, we observed that the first time period (2 weeks of ants foraging on E_low_ and E_high_ leaves) showed the greatest difference between treatments (Figure 1). At the 2 week harvest period, the E_low_ gardens were on average 77% larger (per worker) than the E_high_ gardens (Figure 1, Mann–Whitney U = 55, n = 29, P = 0.03). This pattern was due to both a greater mean number of workers (56 vs. 47) and greater mean fungal garden mass (dry values, 0.20 g vs. 0.08 g) for the E_low_ and E_high_ gardens, respectively.

**Table 1:** General linear models with the independent variable of treatment nested within sample time

**Dependent variable**	**F**	**Degrees of freedom**	***P***	**E**_**low**_**mean (se)**^**1**^	**E**_**high**_**mean (se)**^**2**^
Fresh garden mass	1.49	5,103	0.20	2.55 (0.49)	2.71 (0.46)
Dry garden mass	0.57	5,100	0.72	0.82 (0.17)	0.84 (0.14)
Total no. workers	0.40	5,102	0.85	224 (43)	255 (42)
Queen mass	1.3	5,101	0.26	0.18 (0.006)	0.20 (0.006)
Fresh garden mass per worker	2.14	5,100	0.06	10.7 (0.70)	9.4 (0.66)
Dry garden mass per worker	2.20	5,100	0.06	3.24 (0.28)	2.86 (0.26)

1. The mean ± 1 standard error of each depended variable for colonies feeding on material with low endophyte content, across all sample times.

2. The mean ± 1 standard error of each depended variable for colonies feeding on material with high endophyte content, across all sample times.

### Laboratory vs. field comparisons

The abundance and diversity of culturable bacteria and fungi in the fungal gardens differed between endophyte treatments and also when fungal gardens were sampled from the laboratory versus the field (Table 2). The fungi cultured from garden isolates included the symbiont fungi and also other fungi (non-symbiont) that may or may not have been endophytes. E_low_ gardens showed very high fungal loads of non-symbiont fungus relative to E_high_ gardens. This finding may have been a sign of persistent non-symbiont fungal infection, since the symbiont fungus was significantly less culturable from E_low_ relative to E_high_ fungal gardens (Table 2). If so, it suggests the symbiont fungus was being outcompeted by non-symbiont fungi. We also observed a higher diversity of morphospecies isolates in E_high_ relative to E_low_ fungal gardens (Table 2). Comparing the laboratory and field colonies, we observed significantly more bacteria and fewer non-symbiont fungi in field gardens relative to laboratory gardens. Also, the diversity of non-symbiont fungal morphospecies in garden isolates was significantly greater in field relative to laboratory colonies (Table 2).

**Table 2:** **The mean (± 1 se) values for culturable fungi and bacteria in laboratory and field fungal gardens**

	**Laboratory gardens**		**All laboratory gardens**	**All field gardens**
	**E**_**low**_**n=9**	**E**_**high**_**n=9**	**n=18**	**n=19**
% symbiont fungi^1^	11(5)	51(9) ***	31(7)	34(8)
% non-symbiont fungi^2^	95(6) ***	64(6)	80(6)***	37(5)
Non-symbiont fungal diversity^3^	0.45(0.06)	0.63(0.04)**	0.54(0.04)	0.88(0.03)***
% bacteria^4^	7(3)	9(3)	8(2)	43(4)***

* P<0.05, **P<0.01, ***P<0.001 using a t test.

1. The mean percentage of fungal isolates from the garden that produced a colony of the ants’ fungal garden symbiont.

2. The mean percentage of fungal isolates from the garden that produced a fungal colony that was not the ants’ symbiont. These non-symbionts included fungi known to be endophytes, soil fungi, and parasites of the ants’ symbiont.

3. Diversity was estimated as the number of morphospecies per isolate on a plate with fungal garden isolates. Values range from 0 to 1, with 1 as the highest diversity level in a case where each isolate is a unique morphospecies.

4. The mean percentage of fungal isolates from the garden that produced a bacterial growth.

*A. colombica* survival in the field during the first months was low. At the time of worker emergence, our plots yielded 2, 6, 9 and 36 colonies. After four weeks of foraging, our four plots yielded 0,0,1 and 22 colonies that survived, so that overall (23/375) 6% of the colonies survived after the first month of foraging.

### Molecular identifications

We sequenced the ITS region for several of the most common endophyte morphospecies present in the cucumber leaves given to the leaf-cutter ants (Table 3). We further isolated nonsymbiont fungi from the laboratory gardens and from fungal gardens in the field, and sequenced the most common morphospecies (Table 4). None of most common endophytes were similar to isolates of the most common non-symbiont fungi from the ants’ cultivar (Table 3, Table 4). *Hypocrea virens* (a fungal parasite that is a teleomorph of *Trichoderma virens*) was the top match for all three of the most common morphospecies isolated from the fungal gardens of laboratory colonies. This suggests that our pure cultures representing morphospecies of non-symbiont fungi were not actually pure, and contained *H. virens* from the ants’ fungal colony as well.

**Table 3:** **Top GenBank matches for the five most common endophyte morphospecies isolated from *C. sativus *(cucumber) leaves that were given to leaf-cutting ants during the feeding experiment**

**Morpho-species and Isolates code**	**% of all isolates in collection**	**Top GenBank match**	**%**	**GenBank Accession Number for top match**	**Gen Bank Accession No. for this study**
A (1255)	29	*Xylaria* sp.	99.62	FJ799950	JX997748
A (1260)		*Xylaria* sp.	99.43	FJ799952	JX997749
C (1361)	20	*Colletotrichum gloeosporioides*	100.00	JX231012	JX997750
E (1390)	3	*Pestalotiopsis mangiferae*	99.79	JX305704	JX997751
E (1392)		*Pestalotiopsis clavispora*	100.00	HM999899	JX997752
F (1398)	2	*Annulohypoxylon stygium*	99.80	EU272517	JX997755
F (1399)		*Annulohypoxylon nitens*	94.53	EF026138	JX997753
G(1414)	2	*Cochliobolus kusanoi*	99.79	JN943395	JX997754

**Table 4:** Top GenBank matches for non-symbiont fungal strains isolated from fungal gardens in the laboratory and in the field

**Location of colony**	**Morpho-species and isolates** **code**	**Top GenBank match**	**%**	**GenBank Accession Number for top match**	**Gen Bank Accession No. for this study**
Lab	1 (CON1035)	*Hypocrea virens*	100.00	HQ608079	JX969615
Lab	2 (CON2013)	*Hypocrea virens*	100.00	HQ608079	JX969616
Lab	3 (CON2015)	*Hypocrea virens*	100.00	HQ608079	JX969617
Field	1 (CON4008)	*Bionectria ochroleuca*	99.59	HQ607798	JX969620
Field	1 (CON4011)	*Curvularia affinis*	100.00	GQ352486	JX969625
Field	1 (CON4012)	*Bionectria ochroleuca*	99.59	HQ607798	JX969621
Field	2 (CON4015)	*Hypocrea lixii*	100.00	FJ442609	JX969618
Field	2 (CON4029)	*Hypocrea lixii*	100.00	HQ608036	JX969619
Field	3 (CON4016)	*Purpureocillium lilacinum*	100.00	HQ842835	JX969622
Field	4 (CON4032)	*Staphylotrichum coccosporum*	99.00	AB625586	JX969624
Field	5 (CON5000)	*Neocosmospora vasinfecta*	99.39	L36627	JX969623

## Discussion

We observed a trend toward a greater fungal garden mass per unit worker when the ants were fed a low endophyte diet, but the difference was only marginally significant over the whole experiment. The trend was driven by the colony measurements from the first sampling, two weeks after worker ants emerged and began cutting leaves. A foundress queen rears her first brood of young using trophic eggs, and nourishes the fungal cultivar with secretions [6,7], so we did not provide queens with leaves for cutting. Thus, this result of smaller colonies and fewer workers is solely due to effects of endophytes on workers, not the queen. This suggests that endophyte loads may limit colony productivity for very young colonies with few, naïve workers, but that within a month of cutting the colony productivity (as measured by fungal mass per unit worker) was similar between low and high endophyte treatments.

*H. virens*, a fungal parasite and the teleomorph of *Trichoderma virens*[24], was sequenced from several of our laboratory colony isolates (Table 3). It may have been present in the laboratory colonies at higher levels than in the field colonies. Further, our sampling of fungal gardens after 16 weeks of leaf-cutter ant foraging showed there were greater levels of non-symbiont fungal infection in E_low_ relative to E_high_ laboratory colonies. One possible interpretation is that the presence of high endophyte loads in leaf material brought by the ants may actually help the leaf-cutter ants’ symbiont defend against fungal parasites in the garden. This echoes the hypotheses in human agriculture that polycultural practices help defend crops against herbivores and diseases [25], or that a diverse microbiome should be less susceptible to successful invasion by a pathogen [18]. Interestingly we also observed that fungal gardens freshly harvested from the field had higher bacteria and lower non-symbiont fungal loads than our laboratory colonies, suggesting that the microbial community in laboratory gardens was disturbed and not representative of natural communities.

Our experiment was limited by several factors. First, the laboratory setting protected all colonies from soil pathogens, predators, fungal parasites, and potential fungal mutualists that the ant colonies would have experienced in nature. Second, due to the practical difficulties of producing large quantities of forage material for our experimental colonies, we provided the ants with an artificially narrow diet relative to what they would forage on in nature [9], though colonies near monoculture (human) farms would naturally have lower diversity of forage material. We chose the plants *C. sativus* and *M. esculenta* because they were experimentally tractable, relative to rainforest plant species that would not produce enough leaves rapidly enough to support our colonies. Finally, the levels of endophytes in our E_low_ treatment were higher than we expected with respect to previous experiments where “forest inoculations” were used. For example, in Bittleston *et al.*[17], *Cordia alliodora* leaves with low endophyte treatments resulted in 13% of the leaf area containing endophytes (vs. 39% in this experiment). Thus, the difference between treatments was less than ideal to see whether endophytes play an important role in fungal garden development.

Considering that endophytes are symbionts that obtain resources from their hosts and grow within their hosts, it is plausible that endophytes of woody plants have evolved ways to defend their hosts, and thus themselves, from being eaten or carried away and decomposed. Plants generally defend themselves from herbivore attack using: (i) inducible defense, whereby herbivore attack elicits changes in leaf chemistry that reduce palatability, (ii) constitutive or “quantitative” defense, in which leaf toughness, fiber, leaf-chemistry, and/or low nutrient availability collectively work to increase an herbivore’s development time on a particular food plant (reviewed in [26,27]), (iii) or a combination of inducible and constitutive defense. Constitutive defenses that slow herbivore growth lead to the prediction that longer development time of herbivores will increase their exposure to predators and parasites [26]. We suggest that endophytes are functionally analogous to constitutive defenses toward ants and their cultivar, in that increased development time (slower growth rates) of ant colonies leads to greater rates of mortality among incipient colonies.

Detailed longitudinal studies in natural populations are lacking for most leaf-cutter species, as is true for ants in general [28]. For *Atta capiguara*, fewer than 2% of incipient nests survive through the claustral stage (i.e., when the first workers emerge) [29], but successful nest establishment also depends on the local density of mature colonies. A review of the limited data for *Atta* gives comparable figures for other species [28], although factors other than disease may also be important. In *A. sexdens*, for example, probability of nest survival is also contingent on duration of digging activity [30]. In a savannah species (*A. laevigata*), about 3% of inseminated queens successful established a nest ( [31], cited in [32]). After the first workers emerged, nest mortality was approximately 45% per month for the next 25 months, then monthly mortality rates dropped to single digits [32]. At our study site, where *Atta* spp. are abundant, ~6% of the incipient *A. colombica* queens resulted in productive colonies. Given these mortality schedules, our data raise the possibility that there is a differential cost of endophytes to the colony during the early weeks of establishment—an important time interval for determining colony survival [28]—relative to already-established colonies. Since our laboratory colonies tended to survive this mortality window despite endophyte loads, however, this hypothesis assumes that the cost of endophytes in ant forage material interacts with other environmental or biotic factors to cause less productivity or greater mortality. For example, workers of incipient colonies are monomorphic, while those of mature colonies are highly polymorphic [6,7]. Tiny minor workers, which have relatively larger antibiotic- producing glands, are efficient at removing pathogenic spores from interstices deep within in a garden, and are recruited to sites of infection by larger major workers [33,34], but these size-based efficiencies cannot be realized by a small, monomorphic, work force. Similarly, the production of costly antibiotic compounds and the extra time needed for cleaning leaf material with endophytes, which can increase by 40% on average when leaf material is high in endophyte abundance [16], could decrease the developing colony’s ability to properly patrol and clear the garden from microbial invaders [35,36]. Such an effect would be strongest when there are few workers available to perform colony defensive duties. Accordingly, previous studies have suggested that the number of workers present will influence the colony’s ability to defend itself against pathogens and parasites [37-39].

## Conclusion

In summary, our experiment provided evidence for a cost of endophytes on fungal garden productivity, but only during the first weeks of leaf-cutter ant foraging. Combined with high mortality rates in the field for incipient colonies, this provides partial support for our hypothesis that foliar endophytes defend their host plants from leaf-cutter ant attack. Our alternative hypothesis, that endophyte diversity could actually help colony productivity, was not supported by the measurements of ant colony productivity in this experiment. The microbial communities in fungal gardens, however, did change as a result of the high and low endophyte diets. Whether these changes in the microbial community affect the ant colonies’ defense against pathogens and parasites deserves further attention.

## Competing interests

The authors declare that they have no competing interests.

## Authors' contributions

SV conceived the study, participated in the experimental design, statistical analyses, coordination of data collection and writing of the manuscript; CE participated in the data collection and manuscript preparation; SR participated in the design of the molecular aspects of the experiment and manuscript preparation; FS collected the molecular data and was involved in manuscript preparation; WW participated in the design of the experiment and manuscript preparation. All authors read and approved the final manuscript.

## Supplementary Material

Additional file 1 Additional supporting information on plant inoculation methods. Click here for file
