# Supplementary material for: Leaf endophyte load influences fungal garden development in leaf-cutting ants
Source: BMC Ecol. 2012 Nov 9;12:23. doi: 10.1186/1472-6785-12-23 (PMC3537522; doi:10.1186/1472-6785-12-23)
Supplement: Additional file 1 — Additional supporting information on plant inoculation methods. [file 1472-6785-12-23-S1.doc]

**Leaf endophyte load influences fungal garden development in leaf-cutting ants**

Sunshine Van Bael

Catalina Estrada

Stephen A. Rehner

J. Fabiola Santos

William T. Wcislo

**Additional Supporting Information on plant inoculation methods**

*Laboratory inoculations with endophytes*

*Colleotrichum tropicale* (Rojas *et al.* 2010, strain Q633) was used for leaf inoculation experiments, as it sporulated readily in the laboratory. It is also isolated frequently from many host plants in Gamboa, including *Manihot esculenta* (cassava) (Van Bael *et al.* 2005, Van Bael *et al.* 2012, Van Bael unpublished data).

*C. tropicale* conidia were produced by means of liquid fermentation. For the liquid fermentation, we used 1.5% Molasses Yeast Medium (15g molasses, 2.5g yeast extract, 1 L water)*.* To make the inocula, cultures of endophytic fungi were grown for ten days in Petri dishes with oatmeal agar (18g powdered oatmeal, 12g agar, 600ml H_2_0) until they colonized the entire Petri dish. The Petri dishes were then flooded with 5 ml of sterile water and mycelia and conidia were scraped into Erlenmeyer flasks containing 500 ml of 1.5% MYM. The flasks were put in a shaker at 125 rpm and 23°C for 14 days. Flasks were sealed with foil and parafilm for the initial 7 days and were sealed with cotton (to allow oxygen flow) for the final 7 days.

For inoculations, the contents of the flasks were filtered through a sterile net of nylon stockings to separate the mycelia from the conidia suspension. To concentrate the conidia, the suspensions were centrifuged for 30 seconds, the supernatants were discarded and the conidia pellets were resuspended in sterile water. We added 0.5% Tween 20 to aid in conidia dispersion. Conidia concentrations of 10^7^ - 10^8^ conidia/ml were sprayed onto leaves using a Nalgene aerosol spray bottle (Mean conidia concentration was 9.1 x 10^7^). Conidia concentrations in the final suspensions were determined using a hemacytometer. A control spray was created using sterile water and 0.5% Tween 20. We sprayed *M. esculenta* plants with the conidia suspension or control spray approximately 10 days before providing the leaves to ants.

*Forest inoculations of endophytes*

For the *C. sativus* (cucumber) inoculations, we took advantage of the fact that most sporefall occurs at night in the forest (Gilbert & Reynolds, 2005). As in Bittleston *et al.* (2011), a subset of our plants were taken out of the greenhouse and placed outside 5 nights per week, for at least 10 nights. The plants were sprayed with rainwater and left overnight in fine mesh cages (to protect from insect damage) near the rainforest edge, then returned to the greenhouse benches early the following morning. Low endophyte plants (E_low_) remained in the greenhouse overnight. Thus, all plants were maintained on the same greenhouse benches during daylight growing hours.

*Re-isolations*

We used reisolations of endophytes from leaves to test whether our treatments resulted in significantly greater endophyte abundance and diversity in E_high_ relative to E_low_ leaves. From most leaves that we offered to ant colonies (see sample sizes in results below), we cut a small (~1 cm^2^) section of leaf area to assess the abundance and diversity of endophytes in the leaf material we were offering to the ants. These sections were cut into 30 tiny segments (~1.5 mm^2^ each) that we surface sterilized with 70% ethanol (1 min) and 10% commercial bleach (1 min) (Van Bael *et al.* 2009). Twenty of these leaf segments were plated using sterile forceps on 2% malt extract agar and allowed to incubate for 7 days. For laboratory inoculations of *M. esculenta*, we assessed the proportion of leaf pieces (out of 20) with *C. tropicale* growing out of them after 7 days. For forest inoculations of *C. sativus*, we assessed the proportion of leaf pieces (out of 20) with any fungal endophyte growing out of them after 7 days.

**References for Supporting Information**

1. Bittleston LS, Brockmann F, Wcislo W, Van Bael SA: **Endophytic fungi reduce leaf-cutting ant damage to seedlings**. *Biol Letters* 2011, **7**:30-32.

2. Gilbert GS: **Nocturnal fungi: Airborne spores in the canopy and understory of a tropical rain forest**. *Biotropica* 2005, **37**:462-464.

3. Rojas EI, Rehner SA, Samuels GJ, Van Bael SA, Herre EA, Cannon P, Chen R, Pang JF, Wang RW, Zhang YP *et al*: ***Colletotrichum gloeosporioides* s.l. associated with *Theobroma cacao* and other plants in Panama: multilocus phylogenies distinguish host-associated pathogens from asymptomatic endophytes**. *Mycologia* 2010, **102**:1318-1338.

4. Van Bael SA, Fernández-Marín H, Valencia MC, Rojas EI, Wcislo WT, Herre EA: **Two fungal symbioses collide: endophytic fungi are not welcome in leaf-cutting ant gardens**. *P Roy Soc B-Biol Sci* 2009, **276**:2419-2426.

5. Van Bael SA, Seid M, Wcislo W: **Endophytic fungi increase the processing rate of leaves by leaf-cutting ants**. *Ecological Entomology* 2012, **37**:318-321.
